# Influence of insulin resistance on the development of hepatocellular carcinoma after antiviral treatment for non-cirrhotic patients with chronic hepatitis C

**DOI:** 10.1186/s13027-016-0056-y

**Published:** 2016-02-24

**Authors:** Takeo Hayashi, Eiichi Ogawa, Norihiro Furusyo, Masayuki Murata, Jun Hayashi

**Affiliations:** Department of General Internal Medicine, Kyushu University Hospital, Higashi-Ku, Fukuoka, 812-8582 Japan; Kyushu General Internal Medicine Center, Haradoi Hospital, Fukuoka, Japan

**Keywords:** Hepatocellular carcinoma, Insulin resistance, HOMA-IR, Non-cirrhosis, Pegylated interferon, Ribavirin

## Abstract

**Background:**

Insulin resistance is considered to be an important factor in the progression of fibrosis and the enhancement of the risk of hepatocellular carcinoma (HCC) for chronic hepatitis C patients. The aim of this study was to assess the effect of insulin resistance on the development of HCC by non-cirrhotic chronic hepatitis C patients treated with pegylated interferon alpha-2b (PEG-IFNα2b) and ribavirin.

**Methods:**

This retrospective study consisted of 474 Japanese non-cirrhotic patients with chronic hepatitis C. The cumulative incidence of HCC was estimated using the Kaplan-Meier method, according to insulin resistance by the homeostasis model assessment of insulin resistance (HOMA-IR) and treatment outcome.

**Results:**

The overall sustained virological response (SVR) rate was 45.1 % (214/474, genotype 1: 35.4 % [129/364] and genotype 2: 77.3 % [85/110]). Twenty-one (4.4 %) patients developed HCC during the follow-up period. The 5-year cumulative incidence of HCC of the SVR group (2.6 %) was significantly lower than that of the non-SVR group (9.7 %) (log-rank test: *P* = 0.025). In multivariable logistic regression analysis, HOMA-IR (≥2.5) (hazard ratio [HR] 12.8, *P* = 0.0006), fibrosis status (F3) (HR 8.85, *P* < 0.0001), and post-treatment alanine aminotransferase (ALT) level (≥40 U/L) (HR 4.33, P = 0.036) were independently correlated to the development of HCC. Receiver operating characteristic analysis to determine the optimal threshold value of HOMA-IR for predicting the development of HCC in the non-SVR group showed that the areas under the curve was high (0.80, cutoff value: 3.0). Only three patients (1.4 %) who achieved SVR developed HCC. Two of them had severe insulin resistance and did not show improvement in HOMA-IR after achieving SVR.

**Conclusions:**

Insulin resistance has a strong impact on the development of HCC by non-cirrhotic patients who have PEG-IFNα2b and ribavirin treatment failure.

## Background

Hepatitis C virus (HCV) is a major cause of chronic liver disease, including chronic hepatitis C, which often progresses to cirrhosis and hepatocellular carcinoma (HCC) [1, 2]. The aim of antiviral treatment with interferon (IFN) for HCV-infected patients is not only achievement of sustained viral eradication [3], but also prevention of HCC and liver-related death [4–9]. In spite of recent, marked advances in anti-HCV treatment, HCC is the fifth most common cancer and the third leading cause of cancer-related mortality worldwide [10]. Furthermore, the occurrence of HCC is increasing because of HCV infection, which has infected approximately 170 million people globally.

HCV-infected patients who obtain a sustained virological response (SVR) by IFN monotherapy or pegylated-IFN-alpha (PEG-IFNα) and ribavirin demonstrate significant improvement in liver fibrosis [11, 12] and a decrease in the occurrence of decompensated liver disease and HCC [4–9] compared with untreated or non-SVR patients. Furthermore, male, older age, cirrhosis, non-SVR, and alpha-fetoprotein (AFP) level were reported to be associated with the development of HCC [6, 7].

Among other factors related to the development of HCC, insulin resistance has been reported to be an important factor in fibrosis progression [13] and enhancing hapatocarcinogenesis through multiple pathways with branched-chain amino acid-induced inhibition [14] or oxidative/endoplasmic reticulum stress after postprandial hyperglycemia [15, 16]. Insulin resistance is usually measured clinically by the homeostasis model assessment of insulin resistance (HOMA-IR) [17]. Although we recently reported that insulin resistance undermined the efficacy of HCV clearance in PEG-IFNα2b and ribavirin treatment [18], no reports of the possible association of insulin resistance with the development of HCC after antiviral treatment for non-SVR patients have been provided. This information will be useful for prioritizing patients being considered for treatment with next generation direct-acting antivirals (DAA), especially for non-cirrhotic patients.

The aim of this retrospective study was to evaluate the impact of insulin resistance, by HOMA-IR, on HCC after PEG-IFNα2b and ribavirin treatment for non-cirrhotic patients, based on biopsy or transient elastography (FibroScan).

## Methods

### Patients

This retrospective study consisted of 609 consecutive Japanese patients with chronic HCV infection aged 18 years or older treated with PEG-IFNα2b and ribavirin between December 2004 and November 2010. Patients were enrolled at Kyushu University Hospital, three affiliated hospitals, or one clinic in the northern Kyushu area of Japan (Mitsutake, Yokota, and Haradoi hospitals, and the Kyushu General Internal Medicine Clinic).

The exclusion criteria were: (1) liver cirrhosis measured by biopsy or transient elastography (FibroScan); (2) history of HCC; (3) HCC development during antiviral treatment; (4) PEG-IFNα2b and ribavirin treatment discontinuation; (5) anti-diabetic medication or insulin injection; (6) positivity for antibody to human immunodeficiency virus or positivity for hepatitis B surface antigen; (7) excessive active alcohol consumption (a daily intake of more than 60 g of ethanol) or drug abuse; or (8) other forms of liver disease (e.g. autoimmune hepatitis, primary biliary cholangitis, hemochromatosis). After exclusions, the data of 474 patients was available for analysis. The baseline characteristics of the patients are shown in Table 1. The median follow-up period after the end of treatment was 4.3 (range 0.5–7.2) years. The study was conducted in accordance with the ethical principles of the 2008 Declaration of Helsinki and was approved by the Ethics Committee of Kyushu University hospital.

**Table 1:** Patient baseline characteristics at the initiation of antiviral treatment

Baseline characteristics	
Patient number	474
Age (years)	58 (50–64)
Male, *n* (%)	230 (48.5)
Body mass index (kg/m^2^)	23.1 (21.0–25.3)
Alanine aminotransferase (U/L)	52 (33–81)
Total cholesterol (mg/dL)	174 (152–197)
α-fetoprotein (ng/mL)	4.1 (2.5–7.2)
Platelet count (×10^9^/L)	160 (125–199)
Hemoglobin A1c (NGSP) (%)	5.5 (5.3–5.9)
Fasting plasma glucose (mg/dL)	93 (87–101)
Fasting serum insulin (μU/mL)	8.6 (5.9–13.7)
HOMA-IR	1.9 (1.3–3.3)
HCV RNA level (logIU/mL)	6.2 (5.7–6.6)
HCV genotype, *n* (%)	
1a	4 (0.8)
1b	360 (75.9)
2a	63 (13.3)
2b	47 (9.9)
Fibrosis stage, *n* (%)	
F0-1	241 (50.8)
F2	104 (21.9)
F3	76 (16.0)
Not determined	53 (11.2)
Activity grade, *n* (%)	
A0-1	162 (34.2)
A2	244 (51.5)
A3	15 (3.2)
Not determined	53 (11.2)

Data expressed as number (%) or median (first-third quartiles)

*HOMA-IR*, homeostasis model assessment of insulin resistance; *HCV* hepatitis C virus

### Assessment of clinical and laboratory findings

Baseline clinical parameters included serum alanine aminotransferase (ALT), total cholesterol, plasma glucose, serum insulin, serum AFP, platelet count, and hemoglobin A1c (HbA1c), all measured by standard laboratory techniques at Kyushu University Hospital or a commercial laboratory (SRL Laboratory, Tokyo, Japan) within 1 week before the initiation of treatment. The Japan Diabetes Society (JDS) value of HbA1c was converted for use in this study to a National Glycohemoglobin Standardization Program (NGSP) assigned value by adding 0.4 % [19]. Body mass index was defined as the body mass divided by the square of the height, which is universally expressed in units of kg/m^2^. Clinical follow-up of HCV viremia was done by real-time reverse transcriptase PCR assay (COBAS TaqMan HCV assay) (Roche Diagnostics, Tokyo, Japan), with a lower limit of quantitation of 15 IU/mL and an outer limit of quantitation of 6.9X10^7^ IU/mL (1.2 to 7.8 log IU/mL referred to log_10_ IU/mL) or COBAS Amplicor HCV Monitor Test v2.0 (Roche) using the 10-fold dilution method, with a lower limit of quantitation of 5,000 IU (5 kIU/mL) and an outer limit of quantitation of 5,100,000 IU (5,100 kIU/mL).

### Assessment of insulin resistance

Insulin resistance was measured from fasting samples using HOMA-IR and calculated as fasting serum insulin (μU/mL) × fasting plasma glucose (mg/dL)/405 [17]. HOMA-IR was measured within 1 week before the initiation of treatment. Serum and plasma samples were collected at least 12 h after an overnight fast. Aliquots of fresh serum and plasma samples from each patient were immediately separated and sent at 4 °C to the hospital laboratory for the measurement of serum insulin and plasma glucose.

### Assessment of liver fibrosis

Ultrasound-guided liver biopsy was done by experienced hepatologists within 1 month before initiation of antiviral therapy. The minimum length of the liver biopsy was 15 mm and at least 10 complete portal tracts were necessary for inclusion. For each specimen, the stage of fibrosis was established according to the METAVIR score [20]. The liver cirrhosis of patients with no liver biopsy was diagnosed by transient elastography (FibroScan value ≥14.9 kilopascal; the cutoff value that indicates a negative predictive value for cirrhosis is 100 %) [12].

### Antiviral treatment and patient follow-up

All HCV genotype 1 patients received a combination treatment of PEG-IFNα2b (PEG-Intron; MSD K.K., Tokyo, Japan) and ribavirin (Rebetol; MSD) for 48–72 weeks: The same regimen was done for 24 weeks for genotype 2 patients. Successful treatment was an SVR, defined as undetectable HCV RNA at 24 weeks after the end of treatment. In order to investigate the post-treatment incidence of HCC, the length of the follow-up period was calculated from the end of antiviral treatment to the diagnosis of HCC or the last follow-up visit up to December 2012.

Serum ALT and AFP measurement and abdominal imaging (ultrasonographic examination, or computed tomography) were done every 3–6 months for each patient. The HCC diagnosis was confirmed by needle biopsy, histology of surgically resected specimens, or characteristic radiological findings.

### Statistical analysis

Statistical analyses were conducted using SPSS Statistics version 22.0 (IBM SPSS Inc, Chicago, IL, USA). Baseline continuous data are expressed as median (first-third quartiles) and categorical variables are reported as frequencies and percentages. Univariate analyses were done using the Chi-square, Fisher’s Exact, or Mann-Whitney *U* test as appropriate. Variables with *P* < 0.10 in univariate analysis were evaluated using multivariate logistic regression to identify variables significantly associated with the incidence of HCC. The results are expressed as hazard ratios (HR) and their 95 % confidence interval (CI).

Receiver operating characteristic (ROC) curve analysis was done to evaluate the relationship between the HOMA-IR level and HCC development. The cutoff values were calculated from the ROC curve to maximize the total sensitivity and specificity. Cumulative incidence curves of HCC according to response to antiviral treatment or insulin resistance were plotted using the Kaplan-Meier method, and differences among groups were assessed using the log-rank test. The time frame for HCC incidence was defined as the time from the end of antiviral treatment to the diagnosis of HCC. A *P* value less than 0.05 was regarded as statistically significant in all analyses.

## Results

### Patient characteristics

Of the 474 patients enrolled, 364 (76.7 %) were infected with HCV genotype 1 and 110 (23.2 %) were genotype 2. The median age was 58, and 48.5 % of the patients were men. Almost all (88.8 %) received liver biopsy within the 2 weeks before the initiation of antiviral therapy, with the others diagnosed by FibroScan.

Of the studied patients, 214 (45.1 %) achieved SVR. The SVR rate of patients infected with HCV genotype 1 was 35.4 % (129/364), significantly lower than the 77.3 % (85/110) found for patients with genotype 2 (*P* < 0.0001).

### Development of HCC

Twenty-one (4.4 %) patients developed HCC during the follow-up period. The baseline characteristics of these patients classified by the development of HCC are shown in Table 2. By univariate analysis, the development of HCC was associated with older age (≥60 years) (*P* = 0.0085), higher pre-treatment ALT level (≥40 U/L) (*P* = 0.042), higher post-treatment ALT level (≥40 U/L) (*P* < 0.0001), higher AFP level (≥10.0 ng/mL) (*P* = 0.0030), higher fasting serum insulin level (≥15.0 μU/mL) (*P* = 0.0033), higher HOMA-IR (≥2.5) (*P* < 0.0001), advanced fibrosis (METAVIR F3), and non-SVR (*P* < 0.0001). In multivariable logistic regression analysis, significant independently related factors of HCC were post-treatment ALT level (≥40 U/L) (HR 4.33, 95 % CI 1.09–24.8, *P* = 0.036), HOMA-IR (≥2.5) (HR 12.8, 95 % CI 2.81–93.0, *P* = 0.0006), and fibrosis status (F3) (HR 8.85, 95 % CI 2.99–29.3, *P* < 0.0001). For these non-cirrhotic patients, age and treatment outcome were not extracted as independent factors related to the development of HCC.

**Table 2:** Clinical factors associated with hepatocellular carcinoma

	Univariate analysis			Multivariate analysis		
Parameters	HR	95 % CI	*P* value	HR	95 % CI	*P* value
Age (years)						
≥ 60 (ref. <60)	3.40	1.35–9.68	0.0085			NS
Male (ref. female)	1.77	0.73–4.54	0.21			
Body mass index (kg/m^2^)						
≥ 25.0 (ref. <25.0)	1.28	0.46–4.53	0.65			
Pre-treatment ALT (U/L)						
≥ 40 (ref. <40)	3.12	1.04–13.46	0.042			NS
Post-treatment ALT (U/L)						
≥ 40 (ref. <40)	7.01	2.69–21.76	<0.0001	4.33	1.09–24.8	0.036
α-fetoprotein (ng/mL)						
≥ 10.0 (ref. <10.0)	4.27	1.68–10.5	0.0030			NS
Fasting serum insulin (μU/mL)						
≥ 15.0 (ref. <15.0)	3.99	1.61–9.78	0.0033			NS
HOMA-IR						
≥ 2.5 (ref. <2.5)	15.7	4.47–99.1	<0.0001	12.8	2.81–93.0	0.0006
Fibrosis stage						
F3 (ref. F0-2)	13.9	5.42–40.26	<0.0001	8.85	2.99–29.3	<0.0001
Activity grade						
A2-3 (ref. A0-1)	1.60	0.63–4.56	0.33			
Treatment outcome						
Non-SVR (ref. SVR)	5.23	1.74–22.55	0.0020			NS

*HR* hazard ratio; *CI* confidence interval; *NS* no significance; *ALT* alanine aminotransferase; *HOMA-IR* homeostasis model assessment of insulin resistance; *SVR* sustained virological response

### Overall cumulative incidence of HCC classified by treatment outcome

**Fig. 1:**
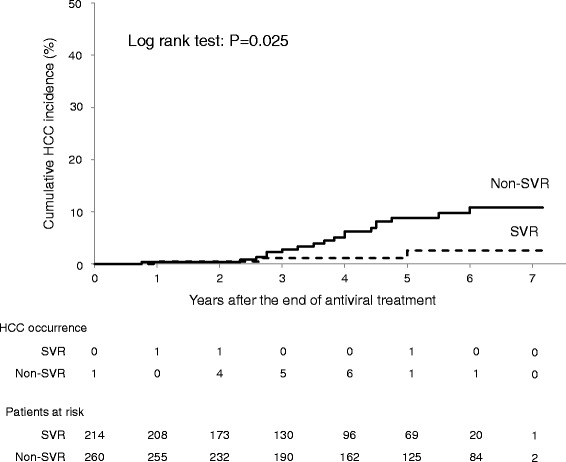
Cumulative incidence of HCC according to treatment outcome (SVR vs. non-SVR) with pegylated interferon alpha-2b and ribavirin. HCC, hepatocellular carcinoma; SVR, sustained virological response

Of the 260 non-SVR patients, 19 (7.3 %) developed HCC, whereas only three (1.4 %) of the 214 patients with SVR did so. The 5-year cumulative incidence rate of HCC of the SVR (2.6 %) group was significantly lower than that of the non-SVR group (9.7 %) (log-rank test: *P* = 0.025) (Fig. 1).

### ROC curve analysis of the effect of HOMA-IR on the development of HCC

ROC curve analysis was done to determine the optimal threshold values of the HOMA-IR levels for predicting the occurrence of HCC in the non-SVR group. The AUROC was 0.80 and the cut-off HOMA-IR level was 3.0 (sensitivity 90.5 %, specificity 73.1 %, positive predictive value 13.5 %, negative predictive value 99.4 %).

### Cumulative incidence of HCC for non-SVR patients, classified by HOMA-IR and post-treatment ALT level

**Fig. 2:**
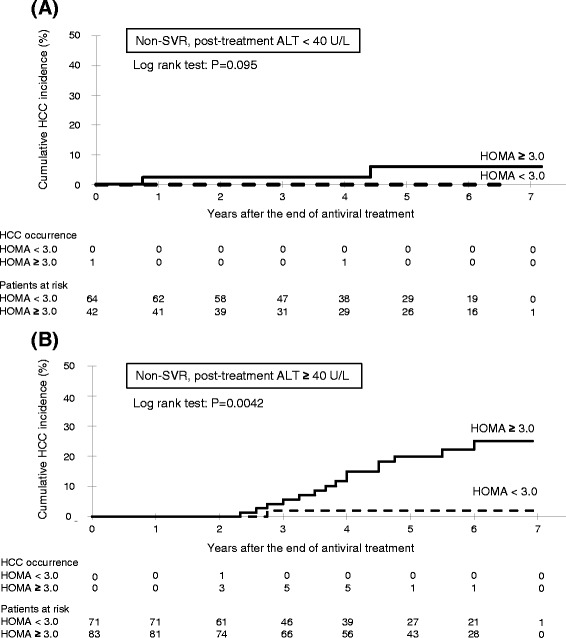
Cumulative incidence of HCC according to insulin resistance (cut-off HOMA-IR 3.0) for non-SVR patients with **a** normal post-treatment ALT level (<40 U/L) and **b** high post-treatment ALT level (≥40 U/L). HCC, hepatocellular carcinoma; HOMA, homeostasis model assessment of insulin resistance; SVR, sustained virological response; ALT alanine aminotransferase

The Kaplan Meyer curves for the estimation of the incidence of HCC by non-SVR patients with post-treatment ALT <40 U/L, classified by HOMA-IR using the 3.0 cut-off level, are shown in Fig. 2a. The 5-year cumulative incidence of HCC in the HOMA-IR ≥3.0 (6.2 %) group was higher, but not significantly, than that of the HOMA-IR <3.0 (0 %) group (log-rank test: *P* = 0.095). The Kaplan Meyer curves for the estimation of the incidence of HCC by non-SVR patients with post-treatment ALT ≥40 U/L, classified by HOMA-IR, are shown in Fig. 2b. The 5-year cumulative incidence of HCC in the HOMA-IR ≥3.0 (22.3 %) group was significantly higher than that of the HOMA-IR <3.0 (2.0 %) group (log-rank test: *P* = 0.0042).

### Cumulative incidence of HCC for non-SVR patients, classified by HOMA-IR and fibrosis

**Fig. 3:**
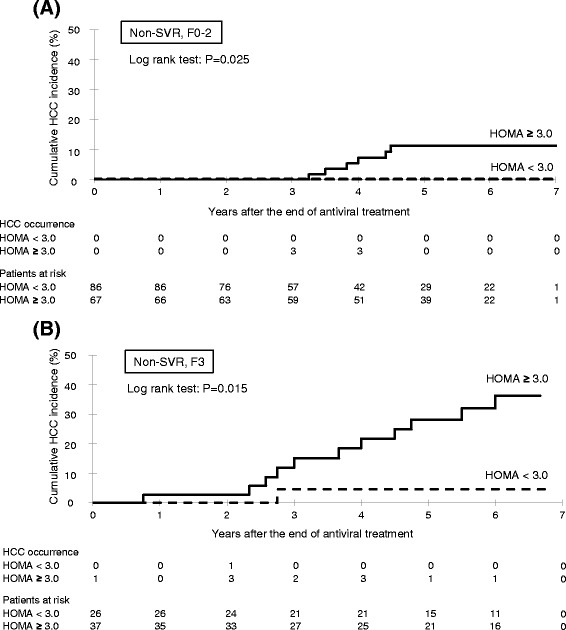
Cumulative incidence of HCC according to insulin resistance (cut-off HOMA-IR 3.0) for non-SVR patients with **a** mild fibrosis (F0-2) and **b** advanced fibrosis (F3). HCC, hepatocellular carcinoma; HOMA, homeostasis model assessment of insulin resistance; SVR, sustained virological response

The Kaplan Meyer curves for the estimation of the incidence of HCC by non-SVR patients with F0-2, classified by HOMA-IR, are shown in Fig. 3a. The 5-year cumulative incidence of HCC in the HOMA-IR ≥3.0 (11.4 %) group was significantly higher than that of the HOMA-IR <3.0 (0 %) group (log-rank test: *P* = 0.025). The Kaplan Meyer curves for the estimation of the incidence of HCC by non-SVR patients with F3, classified by HOMA-IR, are shown in Fig. 3b. The 5-year cumulative incidence of HCC in the HOMA-IR ≥3.0 (32.2 %) group was significantly higher than that of the HOMA-IR <3.0 (4.6 %) group (log-rank test: *P* = 0.015).

### Cumulative incidence of HCC for non-SVR patients, classified by HOMA-IR and AFP level

**Fig. 4:**
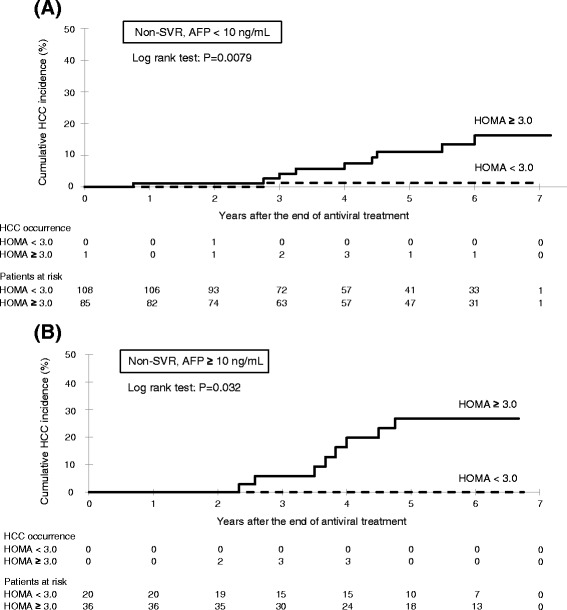
Cumulative incidence of HCC according to insulin resistance (cut-off HOMA-IR 3.0) for non-SVR patients with **a** low to moderate AFP level (<10 ng/mL) and **b** high AFP level (≥10 U/L). HCC, hepatocellular carcinoma; HOMA, homeostasis model assessment of insulin resistance; SVR, sustained virological response; AFP, alpha-fetoprotein

The Kaplan Meyer curves for the estimation of the incidence of HCC by non-SVR patients with AFP <10 ng/mL, classified by HOMA-IR, are shown in Fig. 4a. The 5-year cumulative incidence of HCC of the HOMA-IR ≥3.0 (13.4 %) group was significantly higher than that of the HOMA-IR <3.0 (1.3 %) group (log-rank test: *P* = 0.0079). The Kaplan Meyer curves for the estimation of the incidence of HCC by non-SVR patients with AFP ≥10 ng/mL, classified by HOMA-IR, are shown in Fig. 4b. The 5-year cumulative incidence of HCC in the HOMA-IR ≥3.0 (26.8 %) group was significantly higher than that of the HOMA-IR <3.0 (0 %) group (log-rank test: *P* = 0.032).

### Development of HCC by SVR patients

Of the patients studied, three who achieved SVR developed HCC during the follow-up period, all of whom were infected with HCV genotype 1b treated with PEG-IFNα2b and ribavirin for 48 weeks, without discontinuation. Their fibrosis stage was F3 and two were overweight (BMI ≥25 kg/m^2^) and had severe insulin resistance (HOMA-IR 3.48 and 4.76). However, insulin resistance had not improved adequately at 6 months after achieving SVR (3.25 and 3.77, respectively), irrespective of normalization of the ALT level.

## Discussion

This study of non-cirrhotic patients established important findings by demonstrating a significant association between severe insulin resistance and the development of HCC by non-SVR patients treated with PEG-IFNα2b and ribavirin. It is well known that SVR patients have little risk of HCC [6] and that patients with cirrhosis have the highest risk for the development of HCC. Our data, which show the importance of insulin resistance against HCC development, have clinically useful implications for the management of chronic hepatitis by physicians.

PEG-IFNα and ribavirin has been the standard anti-HCV regimen for the past decade. Although many studies concluded that non-SVR, older age, higher pre/post treatment ALT and AFP levels, and cirrhosis were associated with HCC occurrence, few reports focused on the relationship between insulin resistance and HCC occurrence, especially for non-cirrhotic patients. Indeed, diabetes mellitus enhanced the development of HCC after IFN treatment [21, 22] regardless of treatment outcome, probably because the hyperglycemia increases oxidative/endoplasmic reticulum stress. However, this study mainly consisted of non-diabetic patients (HbA1c median: 5.5 %, range: 4.4–6.8 %), thus, our results that severe insulin resistance, which was in the early stage of impaired glucose tolerance, had enhanced HCC development, irrespective of non-cirrhosis, provide useful information for making a decision on retreatment.

Insulin resistance is usually the main pathology of metabolic syndrome, which is represented by overweight/obesity and a lack of exercise. In contrast, the mechanism of the insulin resistance of patients with chronic HCV infection has been reported to be the harmful effect of HCV core protein [23, 24]. The diversity of the HCV core region has been correlated with ALT elevation and HCC development [25, 26]. In addition, HCV core protein can cause the downregulation of insulin receptor substrate-1 (IRS-1) signaling [23], the elevation of inflammatory cytokine [27], and hepatic steatosis due to impaired secretion of very low lipoprotein cholesterol or decreased fatty acid beta-oxidation [28]. According to a recent report, NS3 and NS5A proteins were significantly associated with the development of HCC [29]. Hyperinsulinemia may play a crucial role as an important factor in the onset or progression of HCC through up-regulation of insulin signal cascades. This could promote fibrogenesis by stimulating the release of connective tissue growth factor, a fibrogenic growth factor from hepatic stellate cells [14]. Moreover, the secretion of various adipokines, such as tumor necrosis factor α (TNFα), interleukin-6 (IL-6), and adiponectin may play a role in the relation between insulin resistance and the development of HCC. Increased production of pro-inflammatory cytokines such as TNFα and IL-6 has been reported to be associated with insulin resistance [30, 31]. On the other hand, adiponectin, which possesses anti-inflammatory and insulin-sensitizing properties, has been positively correlated with the development of HCC [32, 33]. A possible explanation is that adiponectin levels may be increased in cirrhosis and with increasing stages of fibrosis [34]. In any case, the role of adiponectin in chronic hepatitis C is not well understood and the relation between adiponectin and the development of HCC remains controversial.

Based on this study of non-cirrhotic patients, pre-treatment HOMA-IR level, post-treatment ALT level, and fibrosis were independent factors for the development of HCC by patients treated with PEG-IFNα2b and ribavirin. Of note, insulin resistance was more closely related to the development of HCC by non-cirrhotic patients than was treatment outcome. We may need to consider early retreatment to avoid increasing the risk of HCC development, especially for patients with insulin resistance and a high post-treatment ALT level, irrespective of the fibrosis status. Interestingly, some cohort studies have shown that metformin, an oral drug widely used for the treatment of type 2 diabetes that improves insulin resistance, can reduce the risk of HCC [35, 36]. Furthermore, Juan *et al*. recently reported that metformin appeared to have a direct anti-HCC effect in animal models [37].

Our goal is for all patients with chronic hepatitis C to eventually achieve SVR to antiviral treatment. Since 2011, various DAAs have become available for use in the clinical practice setting [38], and DAA only regimens will be the first-line HCV treatment worldwide in the near future. However, waiting for many years for the new DAAs to become available will lead to an increased risk of progressed fibrosis and HCC, especially for patients with insulin resistance. Patients who are at high risk for HCC have the greatest need for antiviral treatment, and it needs to be carried out in as timely a manner as possible.

This study has some limitations. We used HOMA-IR to assess insulin resistance. Although HOMA-IR is one of the most commonly used models for estimating insulin resistance, misclassification is possible when interpreting the results. Among chronic hepatitis C patients without diabetes mellitus, the most commonly cited HOMA-IR cut-off values for insulin resistance (3.0) were reported to have possible misclassification [39]. Particularly, the degree of obesity may have an influence on the overestimation of insulin resistance, with overweight patients (BMI 25.0–29.9 kg/m^2^) likely to be misclassified because of higher odds of false positivity for insulin resistance compared to normal weight patients (BMI <25.0 kg/m^2^), irrespective of ethnicity. Furthermore, obesity itself has been related to increased endogenous insulin secretion, decreased insulin clearance, and increased insulin resistance. The second limitation is the lack of post-treatment HOMA-IR data. According to a previous report that studied mainly Caucasian patients, SVR by HCV genotype 1 patients was associated with reduced insulin resistance (HOMA-IR), but non-SVR was not [40]. However, decreased body weight during IFN treatment is a common adverse effect due to general malaise or a depressive state, especially for elderly Japanese patients [41]. This would have an effect on HOMA-IR values and make it difficult to evaluate the true insulin resistance.

## Conclusion

In conclusion, insulin resistance has a strong impact on the development of HCC. Our findings show that HOMA-IR is a simple and practical biomarker for predicting the development of HCC, particular for non-cirrhotic patients, irrespective of treatment outcome, serum ALT, or AFP level. Our findings will be useful for helping physicians make decisions on the appropriate timing and priority of retreatment.

## Abbreviations

HCV: Hepatitis C virus
HCC: Hepatocellular carcinoma
PEG-IFNα: Pegylated interferon-alpha
SVR: Sustained virological response
AFP: Alpha-fetoprotein
HOMA-IR: Homeostasis model assessment of insulin resistance
DAA: Direct-acting antiviral
ALT: Alanine aminotransferase
HR: Hazard ratio
CI: Confidence interval
ROC: Receiver operating characteristic
